# Skeletal muscle fibers play a functional role in host defense during sepsis in mice

**DOI:** 10.1038/s41598-021-86585-5

**Published:** 2021-04-01

**Authors:** Orlando Laitano, Gerard P. Robinson, Kevin O. Murray, Christian K. Garcia, Alex J. Mattingly, Deborah Morse, Michelle A. King, John D. Iwaniec, Jamal M. Alzahrani, Thomas L. Clanton

**Affiliations:** Department of Applied Physiology and Kinesiology, College of Health and Human Performance, University of Florida, 1864 Stadium Rd, Gainesville, FL 32611 USA

**Keywords:** Immunology, Physiology, Medical research

## Abstract

Skeletal muscles secrete a wide variety of immunologically active cytokines, but the functional significance of this response to in vivo innate immunity is not understood. We addressed this by knocking out the toll receptor adapter protein, Myd88, only in skeletal muscle fibers (skmMyd88KO), and followed male and female mice at 6 and 12 h after peritoneal injection of cecal slurry (CS), a model of polymicrobial sepsis. Because of a previously identified increase in mortality to CS injection, males received ~ 30% lower dose. At 12 h, skmMyd88KO caused significant reductions in a wide variety of pro- and anti-inflammatory plasma cytokines, e.g. TNFα, IL-1β and IL-10, compared to strain-matched controls in both males and females. Similar reductions were observed at 6 h in females. SkmMyd88KO led to ~ 40–50% elevations in peritoneal neutrophils at 6 and 12 h post CS in females. At 12 h post CS, skmMyd88KO increased peritoneal monocytes/macrophages and decreased %eosinophils and %basophils in females. SkmMyd88KO also led to significantly higher rates of mortality in female mice but not in males. In conclusion, the results suggest that skeletal muscle Myd88-dependent signal transduction can play functionally important role in normal whole body, innate immune inflammatory responses to peritoneal sepsis.

## Introduction

Skeletal muscles are able to secrete a variety of cytokines and chemokines upon pathogen exposure^1^. However, whether this response contributes functionally to host defense in vivo is unknown. Skeletal muscle has a great capacity to contribute to endocrine and paracrine signaling during host defense because it makes up ~ 40% of the lean body mass^2^, has an enormous area of interface with the circulation and carries the primary storage reserve for amino acid substrates needed to sustain metabolism, to synthesize cytokines and to support immune cell proliferation^3^. Skeletal muscle senses pathogens via toll like receptors (TLRs) on the sarcolemma^4,5^ and has an abundance of receptors for inflammatory cytokines like TNFα^6^ and IL-1β^7^ that can amplify its immune responsiveness.

To test the hypothesis that muscles are a functional component of host defense, we used a classic immunological approach to interfere with a canonical TLR signal transduction pathway as well as IL-1β receptor signaling, only in skeletal muscle fibers^8^. This was accomplished by conditionally knocking out skeletal muscle myeloid differentiation factor 88 (Myd88) expression. Myd88 is a key adaptor protein in canonical TLR signaling as well as in IL-1β signaling. Though alternative non-Myd88 signaling pathways exist and play important roles in some forms of host defense, “Myd88 is essential for the inflammatory responses mediated by all (membrane bound) TLR family members”^9^. Mice in which global Myd88 expression has been knocked out show essentially no blood cytokine responsiveness to either endotoxic shock or sepsis^10,11^.

Our results demonstrate that suppression of the TLR signal transduction pathway through skeletal muscle Myd88 knockout (Myd88KO) has dramatic effects on reducing circulating cytokine expression during septic shock in both male and female mice. Furthermore, Myd88KO influences the recruitment of neutrophils and other leukocytes into the site of infection and increases the mortality rate in female mice. Studies in skmMyd88KO male mice revealed similar effects on cytokine secretion at 12 h post induction of sepsis and significant changes in immune cell populations in the blood.

## Results

**Figure 1:**
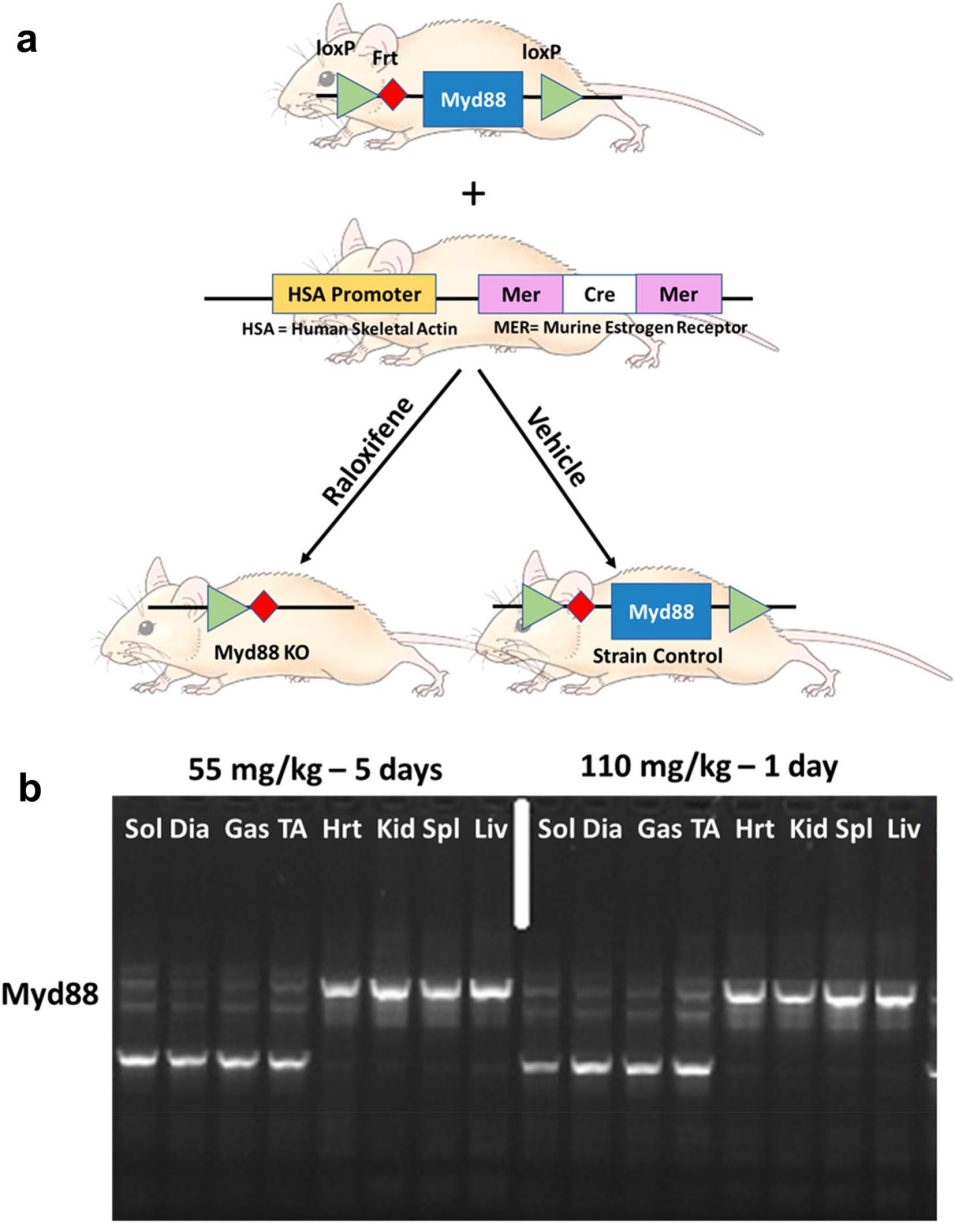
(**a**) Floxed Myd88 (*Myd88*^lox/lox^) were bred with alpha actin merCREmer mice (HSA-*Cre*^*ERT2/−*^) to form homozygote floxed skmMyd88^+/+^ mice with a heterozygote HSA-Cre^Ert/+/−^. Mice were then randomly divided into raloxifene treatment (to induce *Cre*) or vehicle treatment to produce a total of 2 experimental groups. A previous time-matched study reported results of the *Cre* mouse strain (Laitano et al. 2020). (**b**) Genotyping PCR of the skmMyd88KO mice in four representative muscles Sol (slow oxidative soleus), Dia (diaphragm), Gas (gastrocnemius, mixed fiber type) TA (tibialis anterior; fast muscle) and four other tissue types: Hrt (heart), Kid (Kidney), Spl (Spleen) and Liv (Liver). Brightness and contrast adjustments were applied uniformly across all lanes of the PCR gel. Mouse image from https://cliparting.com/mouse-clipart/.

As shown in the schematic in Fig. 1a, homozygote, floxed Myd88 mice were successfully bred with mice carrying the muscle-specific Cre gene, driven by a promoter activated by human skeletal muscle actin and a murine estrogen receptor^12^. The gene, and thus recombinase activity, was successfully induced by treatment with the estrogen analog, raloxifene. There were no significant differences in body weight within male or female mice comparing Myd88KO mice and strain controls (male controls 29.9 ± 10.1 g vs. skmMyd88KO males 29.3 ± 5.4 g; female controls: 23.2 ± 2.6 g vs. female skmMyd88KO: 22.9 ± 3.5 g). There were also no significant differences in body weight changes in response to raloxifene treatment or vehicle treatment during the 4-week recovery prior to sepsis. There were also no significant differences in weight loss or gain during the 6–12 h sepsis exposure period within either sex or group. Genotyping for successful knockout compared favorably to common tamoxifen administration protocols (Fig. 1b). Cre induction and Myd88KO was specific to skeletal muscle tissues when compared to heart, kidney, spleen and liver and showed similar loss of Myd88 DNA sequences to tamoxifen treatments (Fig. 1b). All comparisons in Fig. 1 were made with strain matched sham controls consisting of mice of the same sex and genotype, from the same litters, but treated only with vehicle (Fig. 1a). Parallel experiments in a Cre-induction reporter mouse model (using Gt(ROSA)26Sort^m4(ACTB-tdTomato,-EGFP)Luo/J^)^13^ confirmed specific Cre induction within skeletal muscles and not in other tissues using this approach^14^.

**Figure 2:**
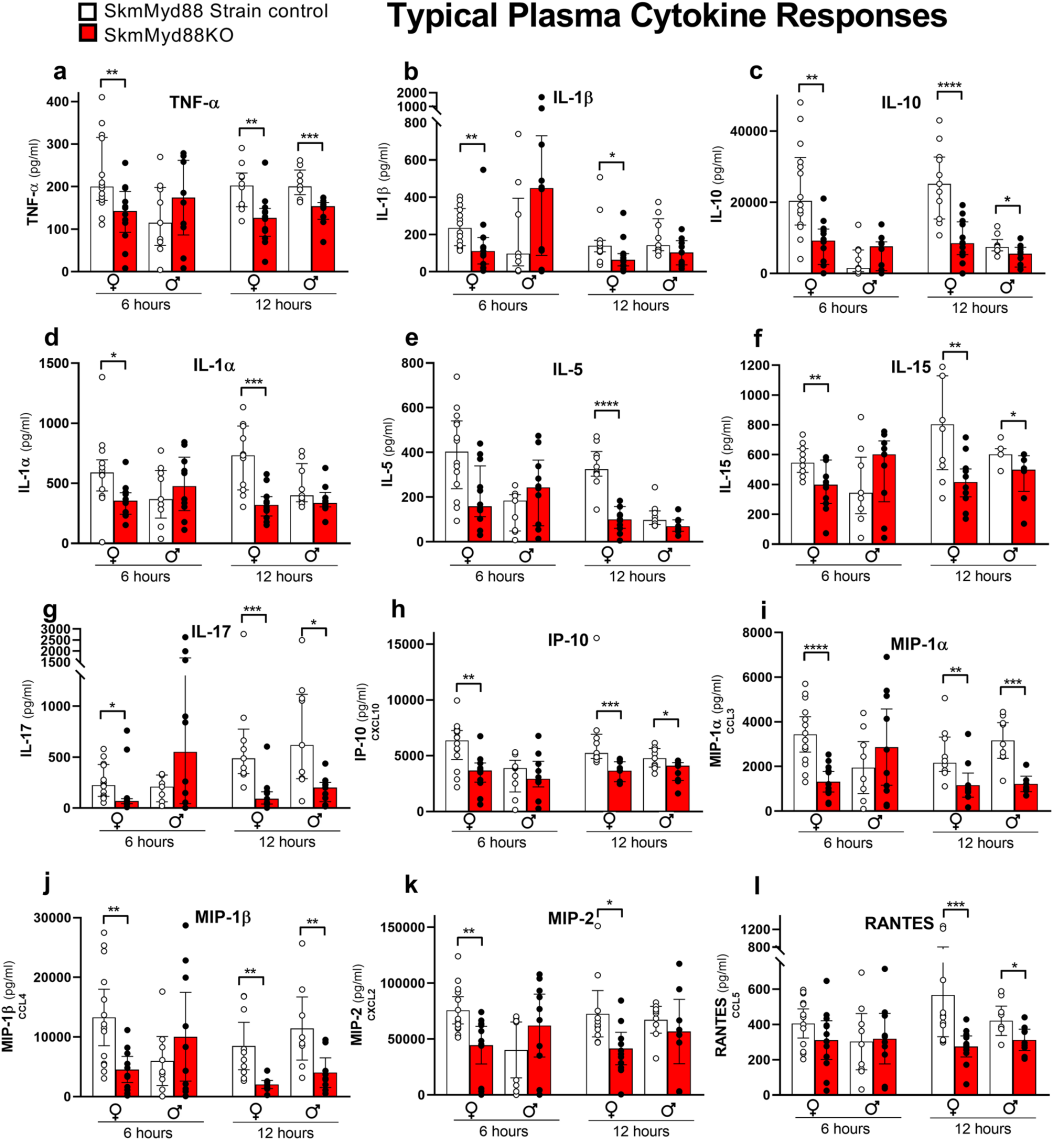
Typical plasma cytokine responses of some cytokines important in sepsis in skmMyd88KO (red) vs. skmMyd88 strain controls (white). Unpaired comparisons were within strains and sexes *P < 0.05, **P < 0.01. n = 9–12 in each group. Data expressed as medians ± 25–75% quartiles. All data met a false discovery rate of < 0.1.

**Figure 3:**
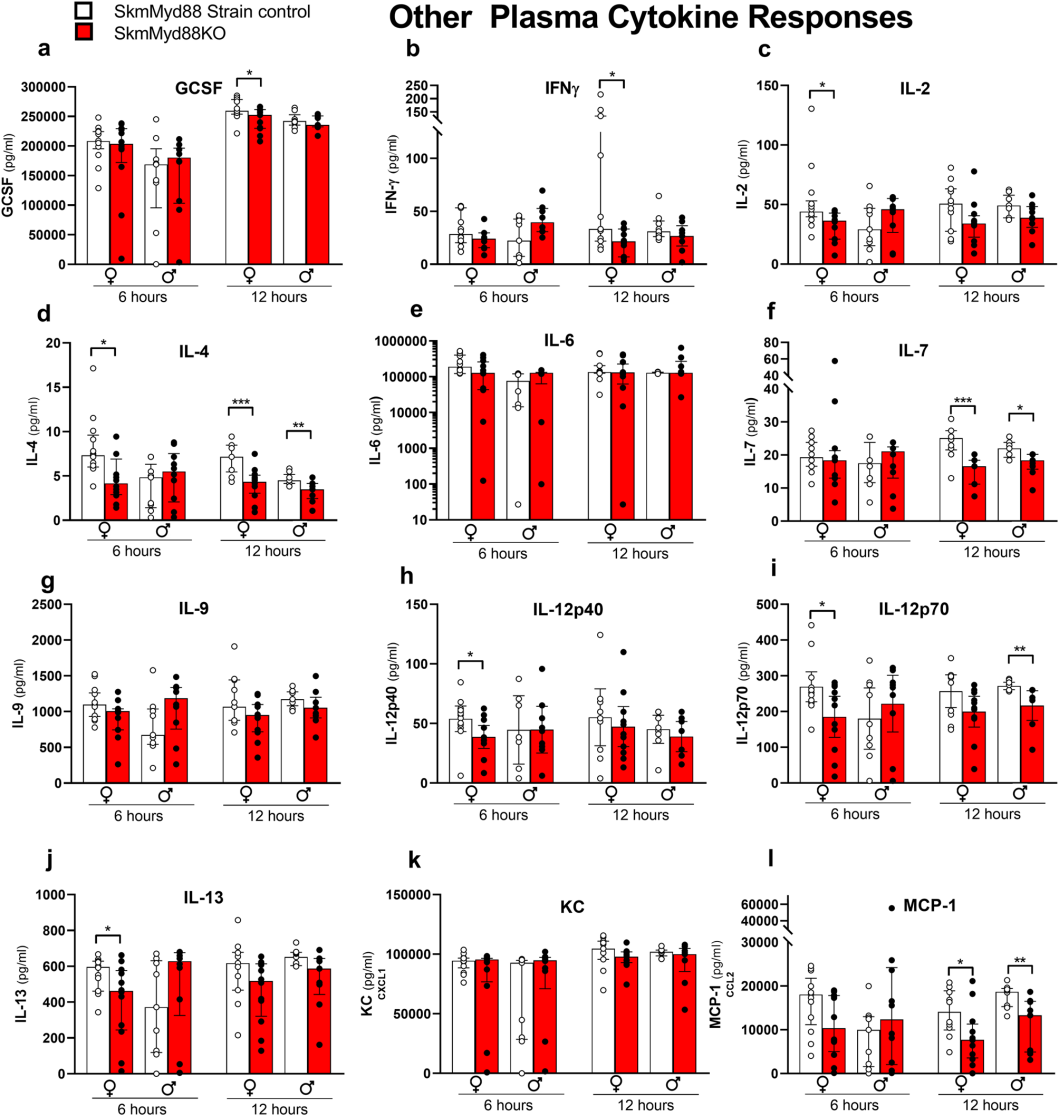
Other cytokine responses to skmMyd88 knockout. SkmMyd88KO (red) vs SkmMyd88 strain controls (white). Unpaired comparisons were within strains and sexes *P < 0.05, **P < 0.01. n = 9–12 in each group. Data expressed as medians ± 25–75% quartiles. All data met a false discovery rate of < 0.1.

Figures 2 and 3 illustrate the changes in measured plasma cytokines in both male and female mice, 6 and 12 h after cecal slurry (CS) injection. Figure 2a–l summarizes the most characteristic cytokine responses across all experimental groups, with the remaining cytokine responses shown in Fig. 3a–l. Most plasma cytokines in females (15/24), measured at 6 h post CS injection, were significantly reduced by skmMyd88KO. The median levels of TNFα (Fig. 2a), IL-1β (Fig. 2b), IL-10 (Fig. 2c), IL-1α (Fig. 2d), IL-15 (Fig. 2f), IL-17 (Fig. 2g), IP10 (Fig. 2h), MIP-1α (Fig. 2i), MIP-1β (Fig. 2j), MIP-2 (Fig. 2k), IL-4 (Fig. 3d), IL-12p40 (Fig. 3h), IL12-P70 (Fig. 3i), and IL-13 (Fig. 3j) were decreased by ~ 1/3 to 1/2 compared to strain-matched controls at this time point. These changes at 6 h were not seen in males. However, at 12 h, post CS injection, many cytokines were significantly reduced by skmMyd88KO in both sexes, including TNFα (Fig. 2a), IL-10 (Fig. 2c), IL-15 (Fig. 2f), IL-17 (Fig. 2g), IP-10 (Fig. 2h), MIP-1α (Fig. 2i), MIP-1β (Fig. 2j), RANTES (Fig. 2l), IL-4 (Fig. 3d), IL-7 (Fig. 3f), and MCP-1 (Fig. 3l). For some cytokines at 12 h, skmMyd88KO impacted only female mice, showing significant reductions in plasma concentrations in IL-1β (Fig. 2b), IL-1α (Fig. 2d), IL-5 (Fig. 2e), MIP-2 (Fig. 2k), IFNγ (Fig. 3b) and GCSF (Fig. 3a). In males, only IL-12p70 was uniquely suppressed by skmMyd88KO (Fig. 3i). There were no effects of skmMyd88KO on plasma IL-6 (Fig. 3e), KC (Fig. 3k) or IL-9 (Fig. 3g) at any time point, in either sex. Overall, these data demonstrate that interference with skeletal muscle fiber Myd88 signal transduction, directly or indirectly, results in marked reductions in the accumulation of cytokines and chemokines in the circulation during the early stages of septic shock.

**Figure 4:**
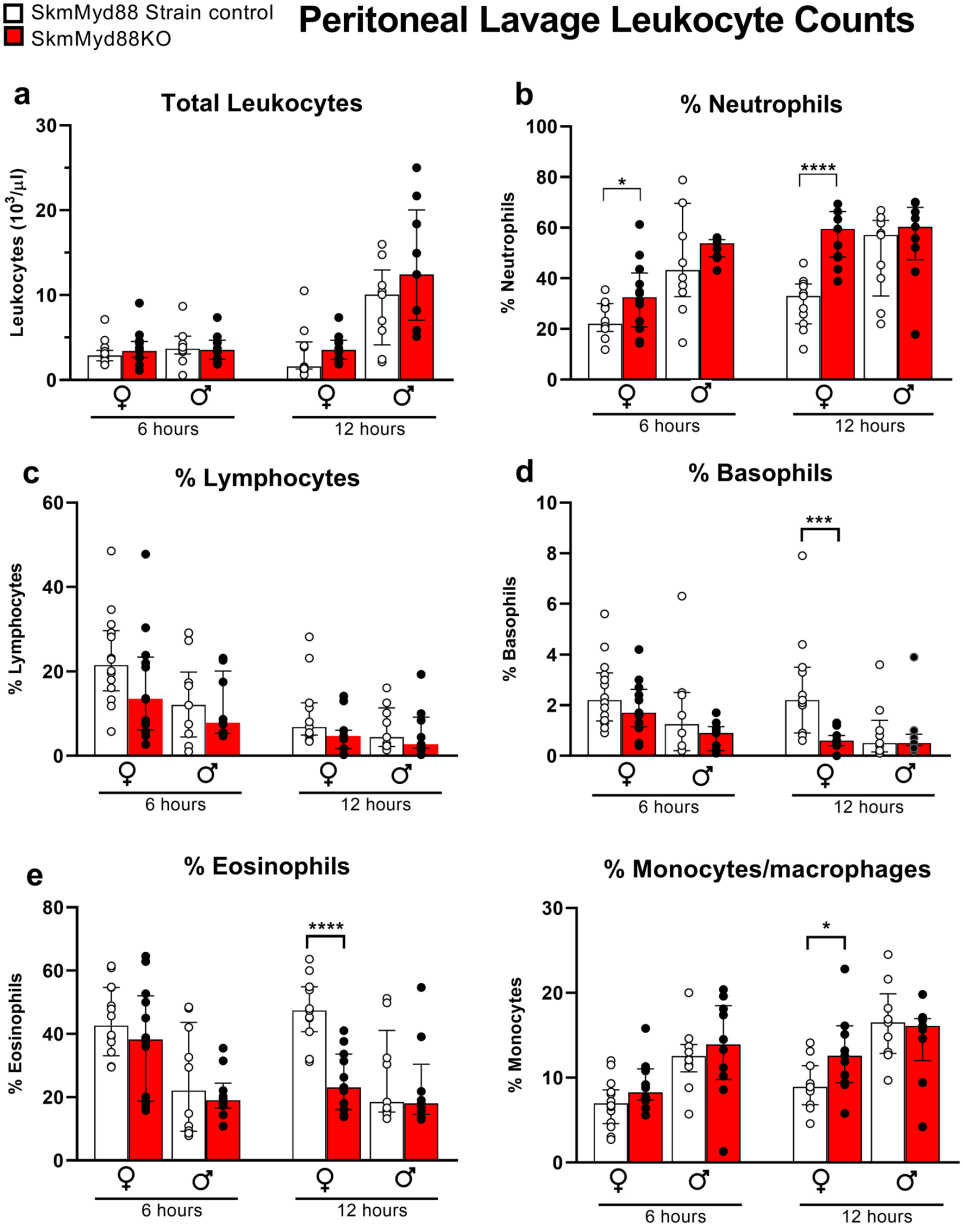
Responses of skmMyd88 knockout on immune cell populations in the peritoneum. SkmMyd88KO red bars, strain matched control, white bars. Groups were tested by orthogonal comparisons within sex and compared to strain matched controls *P < 0.05, **P < 0.01. n = 9–12 in each group. Data expressed as medians ± 25–75% quartiles. All data met a false discovery rate of < 0.1.

Total leukocyte counts in the peritoneum (the site of infection) were not affected by skmMyd88KO in either sex (Fig. 4a). However, at both 6 and 12 h, the %peritoneal neutrophils were elevated by ~ 1.5 and twofold, respectively, in female skmMyd88KO mice (Fig. 4b). In contrast, the %lymphocytes were largely unchanged (Fig. 4c) but the %basophils and %eosinophils were markedly reduced in female skmMyd88KO mice (Fig. 4d,e). The %monocytes/macrophages were increased at 12 h in females (Fig. 4e). No significant changes were observed in differential peritoneal leukocyte recruitment or retention in skmMyd88KO male mice at either time point.

**Figure 5:**
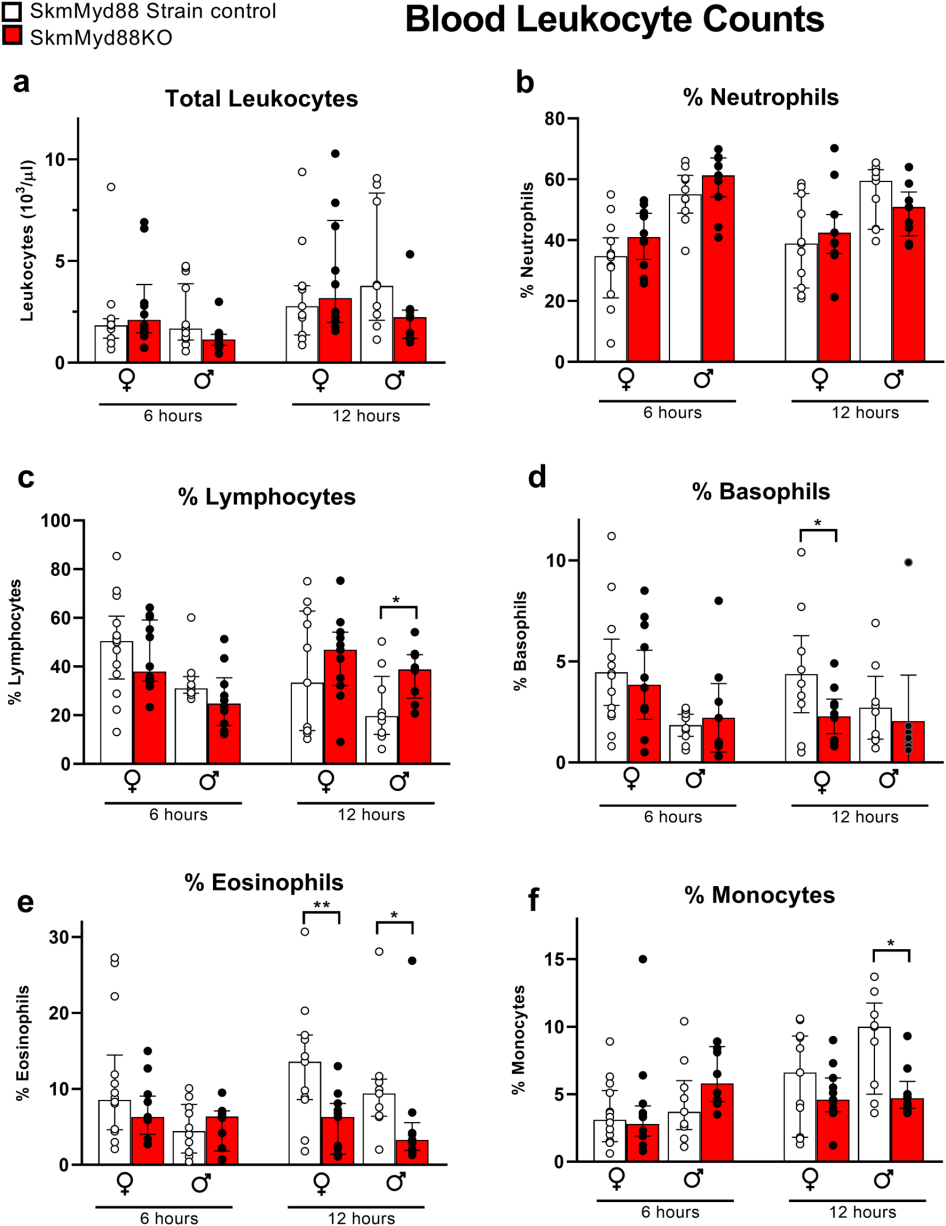
Effects of skmMyd88 knockout on immune cell populations in the blood. SkmMyd88KO red bars, strain matched control, white bars. Groups were tested by orthogonal comparisons within sex and compared to strain matched controls. *P < 0.05, **P < 0.01. n = 9–12 in each group. Data expressed as medians ± 25–75% quartiles. All data met a false discovery rate of < 0.1.

Total leukocyte counts and %neutrophil counts in the blood were not affected by skmMyd88KO (Fig. 5a,b). However, similar to what was observed in the peritoneal lavage, skmMyd88KO mice exhibited decreased %basophils at 12 h in females in blood (Fig. 5d), whereas %eosinophils were reduced by skmMyd88KO in both males and females in blood at 12 h (Fig. 5e). Percent blood lymphocytes (Fig. 5c) were increased and %monocytes (Fig. 5f) were decreased markedly in males at 12 h.

**Figure 6:**
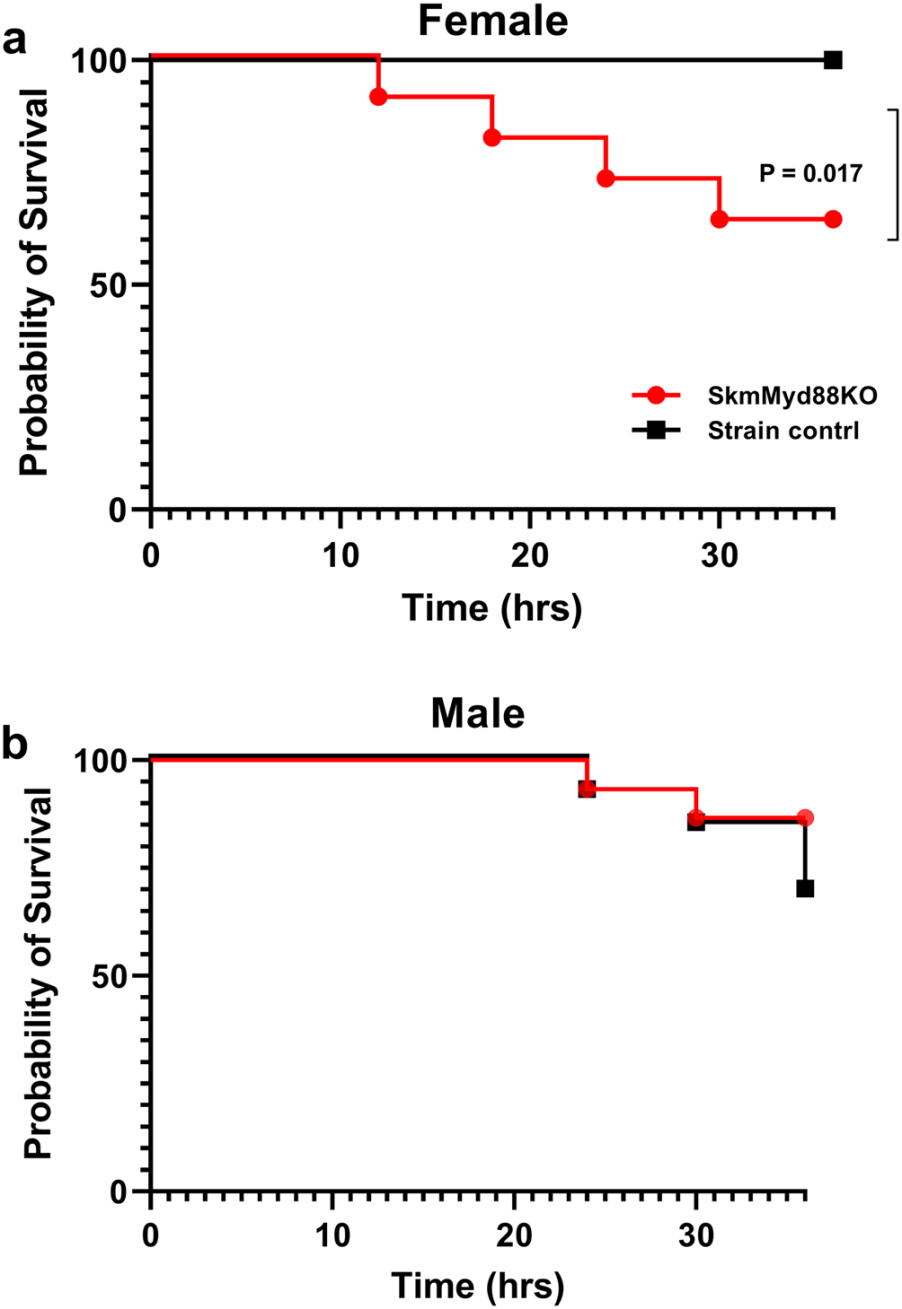
Mortality rates for male and female skmMyd88KO mice compared to strain matched controls. Five day mortality was predicted based on xiphoid temperature measurements at 36 h, as described previously^15^. N = 12 mice per group. P value = Kaplan Meier parametric survival analysis.

Female skmMyd88KO mice exhibited a significantly higher mortality rate (Fig. 6a) compared to strain matched controls, using a highly predictive model (100% specificity) of five day mortality based on noninvasive xiphoid surface temperature changes at this time point^15^. No such effect on mortality was observed in male skmMyd88KO mice (Fig. 6b).

## Discussion

The results demonstrate that skeletal muscle fibers can functionally contribute to host defense by directly or indirectly influencing cell trafficking, cytokine concentrations and mortality. Based on the results of skmMyd88KO, skeletal muscle, in its natural state, must directly or indirectly contribute to secretion of a variety of cytokines and chemokines during sepsis that accumulate in the circulation. This response was most prominent in females but the plasma cytokine responses were also evident in a similar way in males at 12 h. In general, cytokines were suppressed, including broad categories of both inflammatory and anti-inflammatory subgroups. Changes in the trafficking of immune cell populations at the site of infection was most evident in females, and was characterized by a greater movement of neutrophils and monocytes into the peritoneum. Finally, we observed an elevated mortality rate with skmMyd88KO in female mice, but not males, demonstrating that skeletal muscle pathogen responsiveness can play an important role in surviving sepsis under some circumstances.

By knocking out skmMyd88, we expected a partial reduction in the secretion of a limited variety of cytokines that are known to be secreted from skeletal muscle fibers. In this way, we could identify the unique contributions of muscle, in vivo*,* to immune responsiveness. Surprisingly, we found a generalized suppression of nearly all cytokines, an outstanding exception being IL-6, the most recognized muscle-derived cytokine. This was unexpected since not only would suppression of TLR signaling through Myd88 suppress IL-6 transcriptional responses^4^, but reduction of stimuli coming from IL-1β receptor engagement would further reduce IL-6 transcription^16^. Unlike most cytokines, nearly all cell phenotypes in the body are capable of secreting IL-6; therefore, it is possible that other tissues were compensating for loss of IL-6 produced by muscle. In our previous study of skeletal muscle IL-6 knockout during sepsis^14^ we observed only modest effects on plasma IL-6, which supports this view.

Several possible mechanisms may be responsible for the observed global reductions in circulating cytokines in the skmMyd88KO mice during sepsis. (1) The simplest mechanism is that skeletal muscle fibers secrete a significant proportion of most types of circulating cytokines. It follows then that when the ability of muscle to sense pathogens through TLRs is interfered with, it would reduce net cytokine secretions by muscle. This is possible, since muscle is capable of secreting a large number of different cytokines and chemokines during endotoxemia^1^. However, the lack of changes in circulating IL-6 suggests that other mechanisms and tissues are involved and provide larger relative sources of cytokine secretion in this model of sepsis. (2) Muscles contain large concentrations of resident inflammatory cells like macrophages and mast cells as well as myoblasts and satellite cells that lie within the same microenvironment of the muscle fibers^17–19^. By reducing pathogen responsiveness within the muscle fiber microenvironment, the normal paracrine effects of pro-inflammatory cytokines secreted from muscle fibers onto these cells would be reduced, possibly suppressing cytokine responses from these sources. (3) Muscle fibers appear to secrete background anti-inflammatory signals at rest such as irisin^20^, muscarin (osteocrin)^21^ and probably others. In the case of irisin, conditions of sepsis and endotoxic shock drastically reduce this background signal^22^. Therefore, it is theoretically possible that in the skmMyd88KO mice, background muscle-derived anti-inflammatory factors were preserved, thus attenuating global cytokine responses from all other contributing tissues and inflammatory cells. (4) Circulating cytokines may originate largely from activated inflammatory cell populations in both the circulation and at the site of infection. Since some leukocyte phenotypes are changing during the course of infection, the resultant cytokines in the plasma may follow alterations in trafficking and recruitment. We observed that eosinophils and basophils had a strong tendency towards reduction in the blood and in the peritoneum in skmMyd88KO mice, particularly at 12 h. Both eosinophils^23,24^ and basophils^25^ have been shown to strongly contribute to inflammatory cytokine signaling and secretion in murine sepsis. These potential mechanisms are possible, but remain speculative at this time, requiring further study.

The mechanisms behind the altered immune cell trafficking in the peritoneum, particularly in females, could be secondary to the marked changes in circulating cytokines and chemokines or, as discussed above, could drive the changes in cytokines spilling over into plasma. Considering the first possibility, in females we observed a near doubling of the percentage of neutrophils within the peritoneum at both 6 and 12 h time points (Fig. 4B). This may be a result of the accompanying marked reductions in circulating IL-10 to ~ 0.5 and ~ 0.3 of strain-matched controls at these time points, respectively. IL-10 has been shown to be a critical inhibitor of neutrophil recruitment to the site of infection, seen in both IL-10 under-expression and IL-10 over-expression models^26,27^. Furthermore, the effects of IL-10 cannot be overcome by neutralization of TNFα^27^. Therefore, IL-10 is considered a dominant controlling signal for suppressing neutrophil migration into the infection site. The effects of IL-10 on monocyte recruitment are less clear. Since IL-10 inhibits monocyte adhesion to the endothelium^28^, reductions in IL-10 would likely also support recruitment of monocytes into the peritoneum.

The decrement in peritoneal eosinophils may reflect the marked reductions in IL-5 in the circulation (Fig. 3E). In females, eosinophils were reduced to ~ 0. 2 to 0.3 in skmMys88KO compared to strain-matched controls. IL-5 is considered “essential” for eosinophil recruitment from the bone marrow. IL-5 is also known to play a poorly understood, but protective role in sepsis^29^. The reduction in %eosinophils in the blood parallels the reductions seen in the peritoneum (at least in females), suggesting that this may reflect a global influence on eosinophil recruitment. IL-5 affects eosinophil maturation and release from the bone marrow and affects eosinophil survival^30,31^. Basophils, which followed a somewhat similar pattern, are of the same lineage as eosinophils and contain the IL-5 receptor, but the direct effect of IL-5 on basophil populations is not well understood at this time. The only other notable observation was a reduction in blood monocytes at 12 h in the male skmMyd88KO mice. This could reflect movement of monocytes into the peritoneum, thus depleting numbers of blood monocytes.

Another possibility is that recruitment of immune cells into the peritoneum and out of the circulation may be affected by the unique contribution of the diaphragm to leukocyte trafficking into the peritoneum. As discussed in a previous communication^14^, the diaphragm has a unique potential to influence inflammatory cell trafficking into the peritoneum and can be a site of considerable recruitment and adhesion of inflammatory cells^32^. Intraperitoneal fluid exits the peritoneal cavity primarily via stomata on the peritoneal surface of the diaphragm and then into diaphragmatic lymphatic lacunae^33^, eventually draining into the thoracic and abdominal lymphatic nodes and ducts. This means that the diaphragm is exposed to concentrated septic fluid and bacteria from the peritoneum, making the likelihood of TLR stimulation elevated. Secondly, the primary pathway for immune cells to enter the peritoneum is believed to be through “high endothelial venules,” vascular structures within the lymph nodes^34,35^. The network of lymphatic vessels in the diaphragm is in direct communication with the interstitial compartment around the muscle fibers as well as the network of abdominal and thoracic lymphatics and vessels, making the diaphragm well positioned to influence leukocyte trafficking throughout the peritoneum. Interestingly, the changes in immune cell populations in the peritoneum in the skmMyd88KO model (Fig. 4) and the skeletal muscle IL-6 knockout model^14^ during matching sepsis exposures are in the same qualitative direction. This suggests that these results might reflect local changes in IL-6 secretion in the diaphragm affected by either IL-6 knockout or by upstream interference of TLR/Mytd88 signal transduction, known to be a major factor in IL-6 transcriptional regulation.

Although we have discussed above theoretical candidates that could contribute to the effects on cytokine secretion, leukocyte trafficking and mortality, there are many other components of the innate immune network and tissue responsiveness that could have been affected by skmMyd88KO. These include effects on the movement of bacteria or bacterial products into various compartments, the responsiveness of other phenotypes in the body, the half-life of bacteria in the blood or the peritoneum, or other secondary molecular responses within muscle fibers or surrounding cells. However, since the study design allowed us to limit our observations to the effects of downregulation of one protein within the muscle fibers, it seems justified to assign these observations to a functional role of skeletal muscle fibers on innate immunity. This is supported by our previous work knocking out only skeletal muscle IL-6 in this same model^14^.

Male and female responses to skmMyd88KO are difficult to compare directly with this study design because females received higher doses of CS/body weight, based on our experience with dose effects between sexes^15^. The intention was to match the ‘severity’ of infection in each sex rather than dose, such that the dose would be sufficient to result in a ~ 40–60% mortality rate seen in wild type mice^14^. It is possible that the higher dose given to females stimulated a more potent early inflammatory response and therefore a more dramatic early effect of skmMyd88KO in females. Yet another possible factor is the effect of differences in glycerol dosing (15% of the slurry volume) because we gave the female mice a larger dose of glycerol/slurry combination. The immunological effects of intraperitoneal glycerol treatment alone are poorly understood. In future studies 10% glycerol may be a more preferable choice as it has been shown to be effective for slurry preservation^36^. We wish to stress that besides the sex differences in mortality, other male and female immune responses are qualitatively similar. Other biological factors may have also been responsible for sex differences, including the fact that female mice have a greater density of TLRs and a higher responsiveness to LPS challenge in general. These observations have been shown to be estrogen-dependent^37^.

Our method of evaluating mortality in septic mice could also have had some effect on the mortality results (Fig. 6). Early on, while working with the cecal slurry model, the rapid onset of symptoms and the very prolonged suffering over 5–7 days, led our institutional veterinarians to help us refine our methods and develop an accurate (100% specific) prediction model of mortality within 36 h, based on a threshold body surface temperature^15^. Though providing a clear outcome of differences in ‘early’ mortality, there may have been slowly developing causes of mortality in the skmMyd88 strains that we did not observe in wild type mice. Nevertheless, the results clearly show that the female skmMyd88KO mice die more often and more rapidly within the initial 36 h time period.

One concern of the approach used in this study is the possible impact of estrogen receptor activation by administration of the estrogen receptor analog, raloxifene. It is clear that estrogen receptor activation can influence immune responsiveness^38–40^. To avoid these effects, we waited four weeks after raloxifene injection to perform the experiments. The half-life of raloxifene in rodents (rats) is ~ 13 h and is essentially eliminated by four days^41^. However, simultaneous to this study (with the same batch of slurry), to test the potential impact of raloxifene and/or recombinase activation on our outcomes, we directly tested identical groups of mice containing only the Cre gene and its promoter. The Cre mice receiving raloxifene injection demonstrated no significant effects of raloxifene on immune responses or mortality in either males or females^14^. From these results, we concluded that the effects of skmMyd88KO on immune responsiveness were not due to a confounder of raloxifene treatment or recombinase induction. Another concern was that we do not have baseline values for cytokine secretion or leukocyte populations in non-septic mice in these strains. It is possible that the skmMyd88KO vs. its strain control may exhibit some inherent background change in immunological control. However, we would expect these effects to be small and unlikely to explain the large effects seen during sepsis exposure.

What is the overall relevance and implication of our findings? Septic shock is responsible for > 35% of the hospital deaths in the United States^42^. One commonly recognized clinical covariate for poor outcomes in sepsis is low muscle mass^43^. The underlying mechanism for the relationship between low muscle mass and poor outcomes in sepsis is not understood. However, a related factor, regular exercise, appears to also be an important factor. For example, individuals averaging less than the minimum recommended US Federal Activity Guidelines (i.e. ~ 150 min of moderate intensity exercise/week^44^) have ~ twofold higher risk of dying of sepsis in their lifetime^45^. This is supported in numerous animal models where both voluntary and forced endurance exercise training result in striking reductions in sepsis mortality^46–48^. We propose that one component of the apparent effects of a healthy musculature on sepsis outcomes is improved contributions of muscle to innate immune function. However, further work will be needed to test this hypothesis. Understanding the complex ways that skeletal muscle interacts with the integrated immune responses to infection may provide additional opportunities for development of future treatment or prevention strategies to this widespread and deadly disease.

In conclusion, the results suggest that skeletal muscle Myd88-dependent signal transduction is a functionally important component of normal whole body, innate immune inflammatory responses to septic shock. Based on the mortality results in females, these responses can be of sufficient strength to affect the survival of the organism.

## Methods

### Animal care and handling

All protocols for this study were approved by the University of Florida Institutional Animal Care and Use Committee. A total of 128 male and female skmMyd88KO mice were bred in-house. All animals were between 3 and 5 months of age and were housed at the University of Florida Animal Care Facilities, with vivarium temperature and relative humidity maintained between 20 and 25 °C and 31–67%, respectively. Animals were kept under a 12:12 light–dark cycle and had ad libitum access to water and standard chow (2918, Envigo, Indianapolis, IN). All reported information regarding the procedures conformed to the Animal Research Reporting of in vivo Experiments—ARRIVE guidelines^49^.

### Muscle-specific conditional Myd88 knock down

We obtained embryos of floxed Myd88 mice (*Myd88*^lox/lox^) from The Jackson Laboratory (B6.129P2(SJL)-*Myd88*^*tm1Defr*^/J (donated by Anthony L. DeFranco, University of California San Francisco) which were developed on a C57bl/6 background. The mice were bred with a muscle specific inducible *Cre* mouse developed by McCarthy et al.^12^, and provided by Dr. Karyn Esser. The *MER-Cre-MER* construct includes a human skeletal muscle α-actin promoter (HSA) and a mutated estrogen receptor (MER) ligand binding domain to activate the gene, HSA-*Cre*^*ERT2/−*^, also on a C57BL/6 background. Cre expression under this model is specific to skeletal muscle following exposure to an exogenous selective estrogen receptor modulator like tamoxifen or raloxifene. The effectiveness of raloxifene, compared to tamoxifen, to induce tissue specific knockout in our hands was previously tested and reported^14^. Mice were bred to produce both males and females, i.e. Myd88^lox/lox^*Cre*^ERT2/−^, which we will refer to as “skmMyd88.” Separate sets of *Cre*^ERT2/−^ mice, treated and untreated with estrogen agonist, raloxifene to induce recombinase, were studied simultaneously, with results reported in a previous study^14^. Both the Cre and skmMyd88 adult strains were injected intraperitoneally (IP) with either raloxifene (to activate the Cre recombinase) or a matched vehicle injection (no recombinase activation) (Fig. 1A). The raloxifene was administered in a single dose of 110 mg/kg, prepared in polyethylene glycol 400 (PEG400) at a dose of 55 mg/ml, combined in a suspension with equal parts deionized water (DIH_2_O). The vehicle solution consisted of PEG400 and DIH_2_O at the same mg/kg and mg/ml ratios. We performed several trials to determine the appropriate dose and preparation of raloxifene to induce Cre. Effectiveness of raloxifene-induced skeletal muscle Cre activation using this approach was previously demonstrated in our hands^14^ using Gt(ROSA)26Sort^m4(ACTB-tdTomato,-EGFP)Luo/J^ reporter mice^13^.

### Cecal slurry protocol

We induced sepsis using a previously developed cecal slurry model^50^. Slurry for this model consists of the homogenized and diluted cecal contents of many donor mice. This was prepared in a 15% glycerol/ PBS to a concentration of a 100 mg slurry/ml. We aliquoted the slurry into 2 ml cryovials and stored them at – 80 °C in Mr. Frosty freezing containers (Thermo Fisher, Waltham, MA) for later use. We allowed the vials of slurry to thaw at room temperature and vortexed them immediately once thawed before IP injections took place. We injected the animals in the lower right quadrant of the abdominal cavity. All mice were singly housed > 24 h prior to cecal slurry injections. To minimize animal suffering during the experimental procedures, we determined a sensitive and specific method of predicting likelihood of mortality based on surface temperature measured at the xiphoid process^15^ . We determined and reported mortality rates for C57bl/6 males and females at doses ranging from 1.0–1.8 mg/g slurry^14^. For all subsequent experiments within this study, we used a dosage equivalent to 40–60% 5-day mortality for each sex in the Cre strain, which was 1.6 mg/g for females and 1.2 mg/g for males. This approach was an attempt to titrate the level of infection to a percentage of the lethal dose.

### Polymicrobial sepsis and sample collection

For all experiments, mice were injected with cecal slurry between the hours of 0200–0500. We sacrificed the animals for tissue collection at 6- or 12-h post-injection. One hour prior to sample collection, we injected mice subcutaneously with 0.5 ml warm saline to ensure collection of adequate blood samples in mice suffering from shock. At the time of collection, we anesthetized the mice under 1.5–2% isoflurane. To collect the peritoneal lavage sample, we first injected 2 ml of sterile PBS intraperitoneally, followed by a gentle massage of the peritoneal cavity, and then recovered the solution. We then placed the mice into a supine position and collected a blood sample in 0.5 M EDTA from the heart, using a transcardiac stick just left of the xiphoid process. Between 0.5 and 1 ml of blood was collected in most cases. Immediately following collection, whole blood and lavage were analyzed using a hematological analyzer (Heska Element HT5, Loveland, CO) for differential cell counts. We then centrifuged blood samples at 2000×*g* for 10 min at 4 °C, collected the plasma, and immediately snap froze it in liquid N_2_ for storage at − 80 °C. We measured plasma cytokine and chemokine concentrations using a Luminex Magpix® multiplex analyzer (25 Plex MILLIPLEX MAP Mouse Cytokine/Chemokine Magnetic Bead Panel, Millipore, Burlington, MA). We followed manufacturer instructions for sample preparation.

### Experimental design and statistical analysis

Across both time points (6 and 12 h) we ran experiments as matched groups, parallel in time, i.e. skmMyd88 /raloxifene, skmMyd88/vehicle, in each sex. As mentioned, at the same time, Cre matched controls were studied simultaneously and reported previously^14^. For most measurements, groups consisted of 10–12 animals, with both males and females. Initial analysis of variance demonstrated notable differences between the responses of the Cre and skmMyd88 strains, and between males and females, independent of raloxifene treatment. We therefore simplified the statistical approach and focused the analysis within strain, sex and time comparisons. All statistics were performed with SAS JMP Pro® 14.1 (Cary, NC) or GraphPad Prism (San Diego, CA). For each variable, we identified outliers from sample groups where all cytokine measurements were below or above detection limits suggesting a blood sample error or by Dixon Q test for parametric and nonparametric outlier detection^51^ at the two sided, P < 0.01 level. This method detected only very extreme outliers and no more than one sample per group was ever eliminated. Each population was then tested for normality using the Shapiro–Wilk test. When one or more populations were nonparametric, we utilized the Wilcoxon signed-ranks test. Otherwise we used the two-sample T test or Welch’s two-sample T-test for populations of unequal variances, as appropriate. Thus, the data were handled as simple preplanned comparison between matched groups. To avoid errors due to multiple comparison, the P values were tested for significance after Benjamini–Hochberg analysis, using a false discovery rate reported in figure legends. We evaluated mortality using parameterized Kaplan–Meier survival analyses factors with sex, treatment, and crossed effects being included in parameterization analyses.
